# Unraveling Divergent Transcriptomic Profiles: A Comparative Single-Cell RNA Sequencing Study of Epithelium, Gingiva, and Periodontal Ligament Tissues

**DOI:** 10.3390/ijms25115617

**Published:** 2024-05-22

**Authors:** Ali T. Abdallah, Anna Konermann

**Affiliations:** 1 Cluster of Excellence Cellular Stress Responses in Aging-Associated Diseases (CECAD), Medical Faculty and University Hospital Cologne, University of Cologne, 50931 Cologne, Germany; 2 Institute of Medical Statistics and Computational Biology, Faculty of Medicine, University of Cologne, 50924 Cologne, Germany; 3 Cluster of Excellence Cellular Stress Responses in Aging-Associated Diseases (CECAD), Faculty of Mathematics and Natural Sciences, University of Cologne, 50931 Cologne, Germany; 4 Interdisciplinary Center for Clinical Research, University Hospital RWTH, 52074 Aachen, Germany; 5 Department of Orthodontics, University Hospital Bonn, 53111 Bonn, Germany

**Keywords:** epithelial cells, gingival fibroblasts, periodontal ligament cells, single-cell RNA-seq

## Abstract

The periodontium comprising periodontal ligament (PDL), gingiva, and epithelium play crucial roles in maintaining tooth integrity and function. Understanding tissue cellular composition and gene expression is crucial for illuminating periodontal pathophysiology. This study aimed to identify tissue-specific markers via scRNA-Seq. Primary human PDL, gingiva, and epithelium tissues (*n* = 7) were subjected to cell hashing and sorting. scRNA-Seq library preparation using 10× Genomics protocol and Illumina sequencing was conducted. The analysis was performed using Cellranger (v3.1.0), with downstream analysis via R packages Seurat (v5.0.1) and SCORPIUS (v1.0.9). Investigations identified eight distinct cellular clusters, revealing the ubiquitous presence of epithelial and gingival cells. PDL cells evolved in two clusters with numerical superiority. The other clusters showed varied predominance regarding gingival and epithelial cells or an equitable distribution of both. The cluster harboring most cells mainly consisted of PDL cells and was present in all donors. Some of the other clusters were also tissue-inherent, while the presence of others was environmentally influenced, revealing variability across donors. Two clusters exhibited genetic profiles associated with tissue development and cellular integrity, respectively, while all other clusters were distinguished by genes characteristic of immune responses. Developmental trajectory analysis uncovered that PDL cells may develop after epithelial and gingival cells, suggesting the inherent PDL cell-dominated cluster as a final developmental stage. This single-cell RNA sequencing study delineates the hierarchical organization of periodontal tissue development, identifies tissue-specific markers, and reveals the influence of environmental factors on cellular composition, advancing our understanding of periodontal biology and offering potential insights for therapeutic interventions.

## 1. Introduction

Periodontal tissues exhibit a high level of complexity due to the multitude of interacting cells and extracellular matrices, the intimate juxtaposition of soft and mineralized tissues, and the continuous influence of microbial and physical stressors [1].

Within this intricate structure, the oral mucosa stands as the primary barrier against pathogens and is, likely due to this crucial role, one of the most rapidly dividing and healing tissues in our body with a unique scarless healing ability [2]. The epithelium, enveloping the tooth and composing the oral mucosa’s outer layer, forms a direct connection with the tooth surface and originates from the embryonic ectoderm, primarily consisting of epithelial cells [3]. These are morphologically very easy to distinguish from the primary cells of the gingiva as underlying connective tissue, developing embryonically from neural crest cells and mesoderm, predominantly comprising gingival fibroblasts (GF) [4]. Prior research suggests that GFs situated in the subepithelial compartment display notable adaptability. They react to signals emanating from the epithelial layer above, facilitating coordinated responses between epithelial and mesenchymal cells. This interplay serves as a crucial mechanism in maintaining tissue equilibrium both in normal physiological conditions and pathological states [5]. The periodontal ligament (PDL), constituting the third soft-tissue element surrounding the tooth, is a highly specialized fibrous connective tissue. It comprises a diverse array of cell types including fibroblasts, cementoblasts, osteoblasts, endothelial progenitor cells, epithelial cell rests of Malassez, macrophages, osteoclasts, and progenitor/stem cells. Situated between the cementum and alveolar bone, the PDL plays a crucial role in tooth support and maintenance [6]. Serving as the interface between two hard tissue components, the PDL assumes a pivotal role not only in tooth support but also in facilitating tooth nutrition, preserving tissue balance, promoting repair of injured structures, and sensing mechanical forces [7]. Although PDL cells as the predominant PDL-inhabiting resident fibroblastic cell type are thought to control PDL homeostasis and regeneration, it is still unclear which precise cellular subtypes are exactly responsible for these processes [6,8]. PDL cells have a morphologically spindle-shaped appearance very similar to GFs and are additionally localized in close proximity to each other. However, despite their initial visual resemblance, these fibroblastic cells appear to diverge in their functional roles concerning tissue integrity maintenance, response to inflammatory conditions, and participation in immune responses [9]. Studies have also unveiled unique functional activities and distinctive variances in their regenerative and reparative behaviors, which are likely dictated by their disparate expression of specific genes [10]. Utilizing DNA microarray technology, a previous investigation demonstrated that at least one hundred and sixty-three genes exhibit a threefold or greater difference in expression between PDL cells and GFs [9]. These differential gene expression profiles likely underlie the intrinsic functional disparities between the two cell populations and their tissue-specific actions, although comprehensive data remain elusive.

Several crucial factors certainly influence the analysis of oral tissues, including their inherent heterogeneity and the diverse in vivo conditions they encounter, which collectively shape their characteristics. As a result, the intricate nature of these tissues along with the multitude of cellular types composing them contributes to the fact that many aspects of periodontal physiology and pathology remain inadequately elucidated by current research efforts.

This underlines the necessity of acquiring deeper insights into periodontal pathophysiology by defining cell types. Prior to investigating the regulatory dynamics governing functional processes, the refined understanding of tissue and cell composition enabled by evidence-based insights into gene expression patterns would mark a notable advancement in periodontal research. Subsequently, provided with this knowledge, researchers can analyze complex issues such as the underlying mechanisms regulating periodontal functions such as mechanosensitivity, proprioception, protection against trauma, and infection control. To bring periodontal research into a new era, it is of utmost importance to employ more comprehensive experimental approaches capable of analyzing the full spectrum of genes expressed in tissue while also uncovering expression profiles of individual cell clusters and single cells. This dual approach ensures a thorough understanding of periodontal dynamics, spanning both global scale and micro-level.

Next-generation sequencing (NGS) technology has revolutionized transcriptome analysis due to exceptional throughput, depth, and unprecedented accuracy. These advances improve the understanding of gene expression specificities, regulatory mechanisms, and cellular networks, thus ushering in a new era of research [11]. NGS was the initial method of analyzing the entire genome of cell populations such as cultures, tissues, or organs, providing insights into their ‘average genome’. However, recent advances now also enable single-cell RNA sequencing (scRNA-seq) as a groundbreaking advancement, as this method allows the identification and decoding of genetic patterns at the single-cell level while capturing the cellular diversity and complexity of cell populations [12,13]. ScRNA-seq facilitates the parallel analysis of millions of cells as much as the decoding of their transcriptomes and molecular profiles, which are then subjected to rigorous mathematical analysis [14].

In this study, our aim was to gain a deeper understanding of the genetic composition of PDL, gingiva, and epithelium as the three most important oral tissues utilizing cutting-edge scRNA-seq technology and to investigate these different populations in their highly organized complexity and individuality in detail. Our research goal is to decode, characterize, and map these three oral cell types based on their genetic similarities and differences to provide insights into the cellular architecture and underlying mechanisms within the oral environment.

## 2. Results

### 2.1. Dimensionality Reduction and Clustering

**Figure 1:**
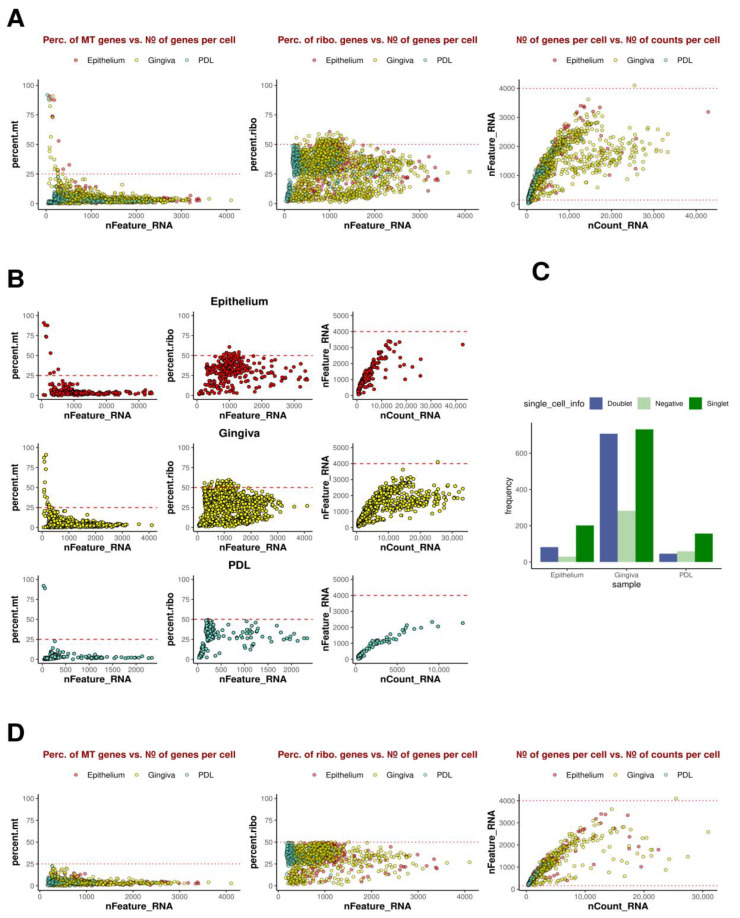
Quality Control Analysis for scRNA-Seq data. (**A**) Consolidated summary of included and excluded cells from tissue samples of epithelium, gingiva, and PDL. The graph on the left illustrates the percentage of mitochondrial genes in the detected transcriptome of each cell (percent.mt), providing information on cell degradation and death, plotted against the number of detected genes in the cell (nFeature_RNA). In the middle graph, the percentage of ribosomal genes, associated with cell cycle phases, is plotted against the number of detected genes (nFeature_RNA). The right graph represents the number of detected genes (nFeature_RNA) plotted against sequenced fragments (nCount_RNA), indicating potential doublets. (**B**) The graphs feature the analyses (**A**) for epithelium, gingiva, and PDL separately. (**C**) Results of HTO demultiplexing, identifying demultiplexed singlet, doublets, and negative signal singlets and their corresponding distribution in epithelium, gingiva, and PDL. All tissue types investigated exhibited the highest frequencies of singlets compared to doublets and negative cells, underlining the quality of the technical procedures. (**D**) Graphical illustration of the post-filtering status of tissue samples after removal of doublets and other biased data, thus enhancing the integrity of subsequent analyses.

**Figure 2:**
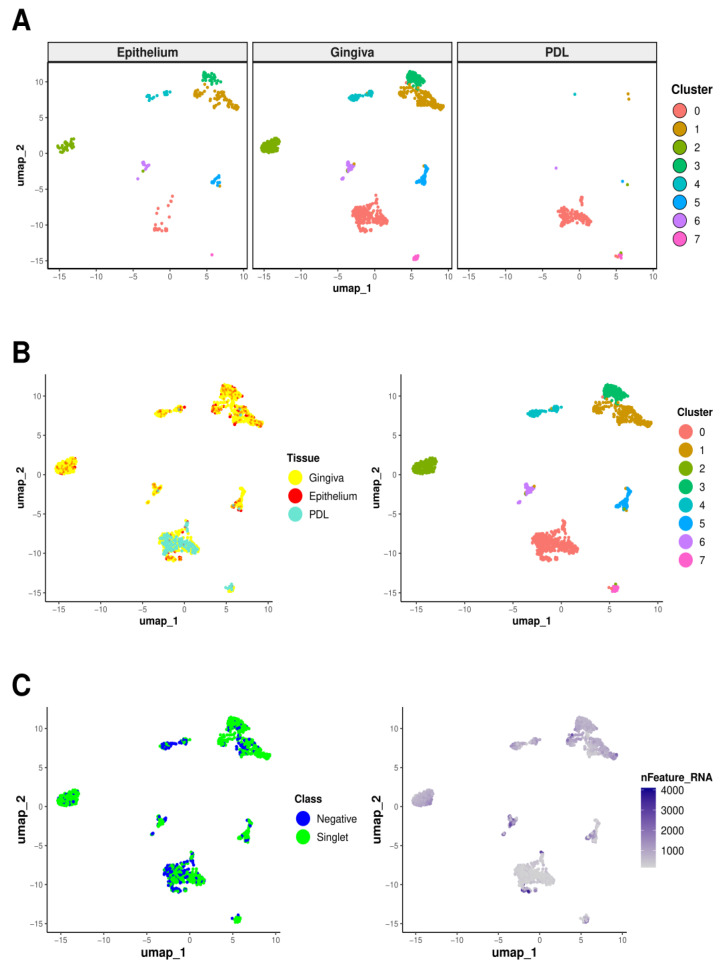
Cluster distribution of tissues via UMAP. UMAP visualization displaying the outcomes of clustering cells derived in vivo from epithelial, gingival, and PDL tissues. Clustering was exclusively based on the biological characteristics of the cells. Each dot on the graph represents an individual cell. Clustering was performed after the integration of the samples, resulting in eight clusters that highlight the distinct cell populations within the integrated dataset. (**A**) UMAPs for each tissue type analyzed, showcasing the tissue-specific distribution of clusters. Cluster 0, harboring most cells compared to the other clusters, predominantly consisted of PDL cells, followed closely by gingival cells. Cluster 7 also exhibited a high percentage of PDL cells, followed by gingival cells, while other clusters had negligible PDL cell numbers. Epithelial and gingival tissues were consistently represented in all clusters. Epithelial tissues predominated in cluster 1, followed by clusters 3, 4, and 6, while cluster 5 has a roughly equal distribution of epithelial and gingival cells. (**B**) The graph on the left groups cells through different coloring by their tissue identity, namely PDL (turquoise), gingiva (yellow), and epithelium (red). The graph on the right groups cells by clusters without splitting by tissue type, providing a general overview of the clustering pattern for the three tissues pooled together. (**C**) In the left graph, cells are grouped by hashtag class into demultiplexed cells (singlets, green) and unassigned cells (negatives, blue). The graph on the right depicts clusters visualized based on the number of genes expressed per cell, with color intensity indicating the magnitude of gene expression per cell, as specified in the legend.

**Figure 3:**
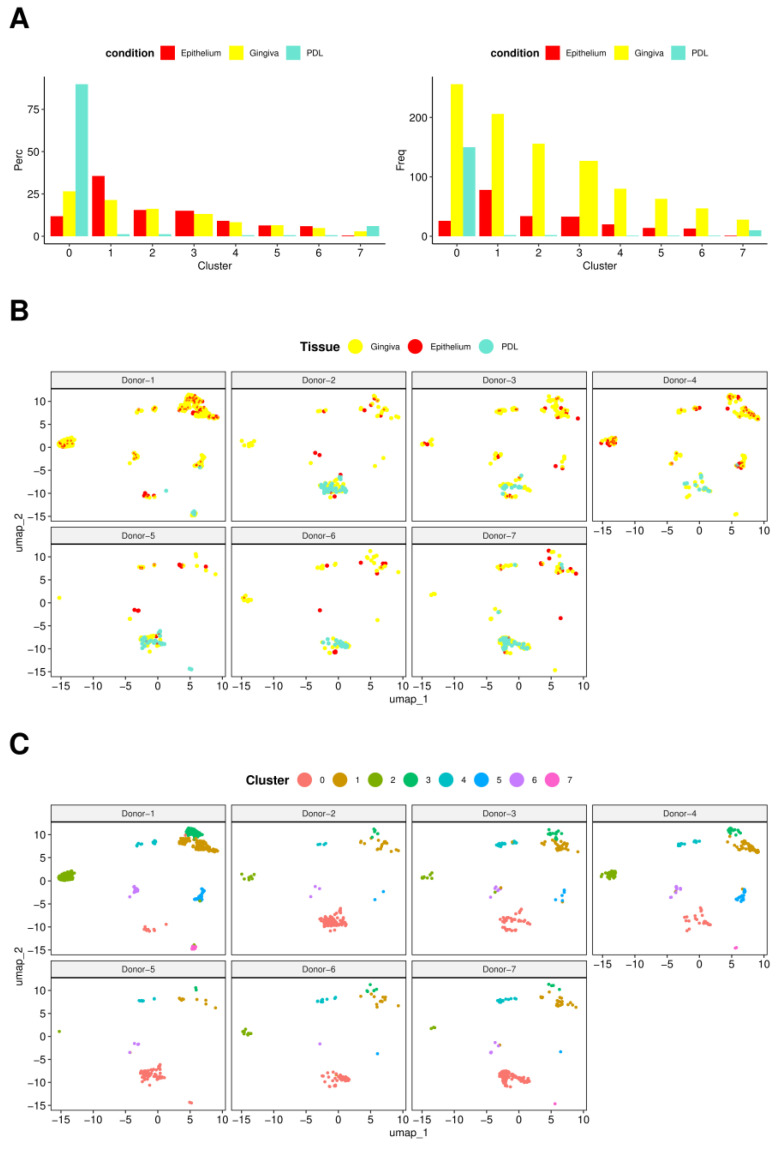
Analysis of cluster Composition across conditions and donors. (**A**) Cell abundance analysis. Bar charts derived from the sample cell abundance analysis exhibit computed clusters by percentage (Perc) and absolute number of cells (Freq), showcasing the distribution of cells from different tissues (epithelium, gingiva, PDL) across clusters. The left graph elucidates the percentage composition of cells (Perc) from each condition within identified clusters. Each bar represents the proportion of cells from a specific condition, ensuring that the total percentage for each condition across all clusters sums up to 100%. Meanwhile, the right graph depicts the absolute number of cells (Freq) from each condition within the clusters, furnishing an exact count that complements the percentage distribution. (**B**) Overview of the donor-specific distribution across samples (top row of UMAP plots) and (**C**) clusters (bottom row of UMAP plots). Cluster 0 was consistently observed across all donors but less pronounced in donors 1 and 4. Donors with a strong presence of Cluster 0 tended to have underdeveloped Clusters 1–3. Clusters 1, 2, and 3 showed significant representation in donors 1 and 4, and to a lesser extent in donor 3.

Following quality control analysis of scRNA-Seq data (Figure 1), cells were clustered using Seurat’s default integration workflow [15], identifying eight distinct clusters. The resulting bar charts and UMAP plots for epithelium, gingiva, and PDL are depicted in Figure 2 and Figure 3.

In Figure 3A, bar charts display how cells from three tissues were spread across clusters. In the left chart, each bar represents the percentage of cells from PDL, gingiva, and epithelium within identified clusters, with all bars totaling 100% for each condition across all clusters. This visualization illustrates how much each of the three tissues contributes to the total cell population within each cluster. A higher percentage in a specific cluster could suggest a strong connection of the tissue type with the cellular population in that cluster. The right bar chart demonstrates the frequency of cells from each tissue within each cluster, offering insights into the absolute size of each cluster and facilitating the comparison of cell numbers from each tissue contributing to each cluster.

Cluster 0 emerged as the predominant cluster both in terms of cellular quantity and representation across all three tissue types. Notably, it exhibited the highest proportion of PDL cells, with gingival cells following closely behind. Conversely, cluster 7 displayed the next highest percentage of PDL cells, again followed by gingival cells, while the remaining clusters exhibited negligible PDL cell numbers. Interestingly, PDL was entirely absent in cluster 3. On the contrary, epithelium and gingiva were consistently represented in all clusters.

Epithelial tissue predominated in cluster 1, subsequent to clusters 3, 4, and 6. Cluster 5 demonstrated a roughly equal distribution of epithelial and gingival cells, while gingival cells predominated slightly in cluster 2. Gingival cells exhibited the highest cell frequency across all clusters, explainable due to their prevalence in isolation procedures regarding the highest cell numbers.

Clusters 0 and 1 harbored the most cells, with cluster 0 displaying either the highest or lowest gene expression level.

Figure 3B shows the donor specificities regarding the representation of the 8 individual clusters in terms of occurrence and characteristic patterns, illustrating the specific distribution of PDL, gingiva, and epithelium tissues for each specimen across all clusters. The diverse weights and distributions of cells from these tissues within and across the clusters highlight inherent inter-donor variations. Notably, the distinct representation and distribution pattern of the eight identified clusters among donors were apparent.

Cluster 0 was consistently observed across all donors and exhibited the highest degree of prominence, except in the case of donor 1, where it was distinctly less pronounced, followed by donor 4. Notably, clusters 1, 2, and 3 exhibited significant representation in these two donors, a trend also observed to a lesser extent in donor 3, presenting a likewise overall pattern across clusters 0–3. Conversely, in donors where cluster 0 showed strong presence, clusters 1–3 were notably underdeveloped. Clusters 4 and 6 were identified in all donors, albeit with relatively low intensity. Cluster 5, however, was entirely absent in donors 5, 6, and 7, and minimally present in donor 2 with only 2 cells. Interestingly, cluster 5 displayed significant prominence again in donors 1, 3, and 4, consistent with the distinct cluster pattern observed. Cluster 7 was solely detected in donors 1, 4, 5, and 7, albeit with a minimal cell count of 1–2 cells. Notably, no discernible correlation was observed between the age, sex of the donors, and the described expression patterns.

Clusters 4 and 6 were identified in all donors with low intensity. Cluster 5 was not detectable in three donors and cluster 7 in four donors. No correlation could be observed between donors’ age, sex, and the individual expression patterns of clusters.

### 2.2. Marker Identification across Clusters

Following cluster generation, markers were identified to elucidate the characteristics and composition of the tissue samples investigated. This involved a dual strategy, conducting a differential expression analysis first and subsequently ranking markers based on their specificity to each cluster.

**Figure 4:**
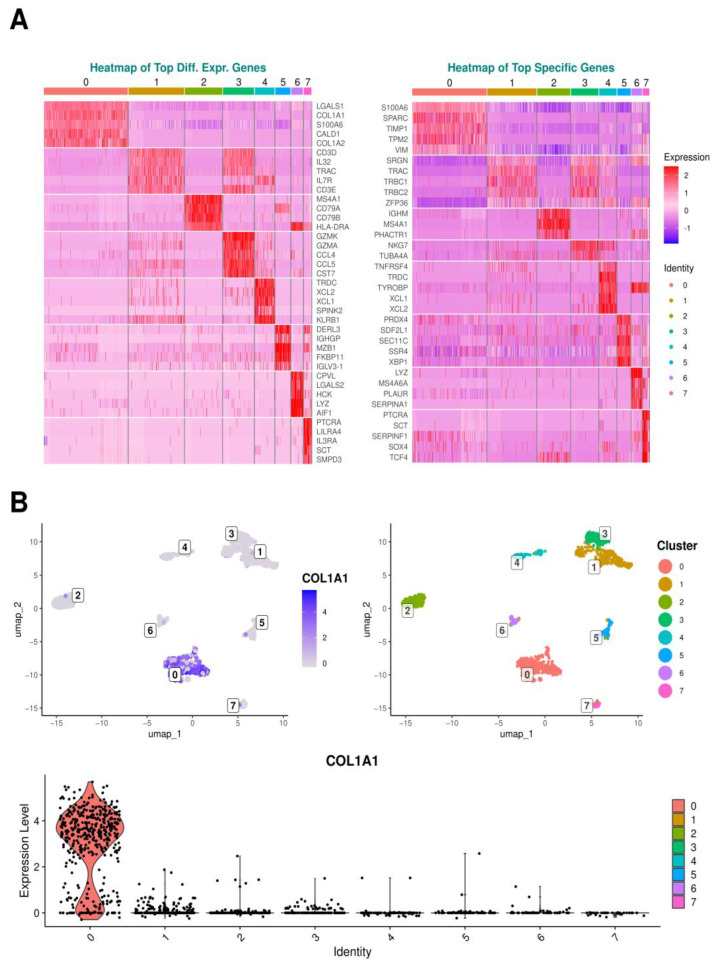
Genetic Profiling and Expression Intensity in Clustered Cells. (**A**) Heatmaps illustrating the gene expression profiles outlined for each cluster. Expression levels are depicted on a color scale from dark blue (low expression) to red (high expression). The heatmap on the left presents the top differentially expressed genes (DEGs) showing the top five differentially expressed markers of each cluster compared to all other clusters. The heatmap on the right shows the top specific genes uniquely characteristic for each cluster, further facilitating the identification of distinct cellular identities. (**B**) The—UMAP plot on the left displays the eight identified clusters for the tissue types PDL, epithelium, and gingiva pooled together and the cluster-specific gene expression pattern of *COL1A1*, with a color gradient indicating the expression levels. The default UMAP plot on the right showcases the eight corresponding clusters. Below, a violin plot details the expression levels of *COL1A1* across clusters, illustrating the distribution and prevalence of expression within each cluster.

Differential expression analysis identified genes with significant expression differences between a particular cluster and all others taken together, shedding light on functional markers potentially important across various clusters. Conversely, specificity ranking focused on markers unique to each cluster, distinguished by their consistently lower or even absent expression in other clusters, potentially revealing more definitive cellular identity signatures. This integrated methodology provided nuanced insight into cluster specificity by revealing both the relative differential expression and the unique expression patterns of genes within clusters. Figure 4B illustrates this aspect by presenting a Uniform Manifold Approximation and Projection (UMAP) plot as much as a violin plot for *COL1A1* expression, one of the prominent differentially expressed genes (DEGs) in cluster 0, showcasing its expression profile across all identified clusters. The analysis examined the most highly expressed genes for each cluster, regardless of tissue type, to determine the genetic specificities of each cluster. Corresponding heatmaps are presented in Figure 4A.

Analyses revealed that the three predominant DEGs in cluster 0 were *LGALS1*, which encodes the cell proliferation-regulating protein galectin-1, followed by *COL1A1*, coding for alpha-1 type I collagen, the primary form of collagen in the body, and thirdly *S100A6*, a gene coding for a calcium-binding protein involved in regulating cell cycle progression and differentiation. The three top specific genes for cluster 0 included, again, *S100A6*, *SPARC* regulating cell–matrix interactions and migration, crucial for wound healing and bone metabolism, and *TIMP1*, an inhibitory molecule inter alia regulating matrix metalloproteinases (MMPs), and playing a pivotal role in extracellular matrix (ECM) composition and wound healing.

In cluster 1, the top three expressed DEGs were *CD3D*, encoding a protein integral to the T cell receptor/CD3 complex essential for T cell development and signaling, followed by *IL32*, which codes for the eponymous pro-inflammatory cytokine and T Cell Receptor Alpha Constant *TRAC*. The three most prominent specific genes for this cluster were *SRGN*, responsible for producing serglycin, a proteoglycan found in endothelial cells, *TRAC* again, and *TRBC1*, which encodes T cell receptor beta constant 1.

Within cluster 2, the primary DEGs included *MS4A1*, coding for a B lymphocyte surface molecule involved in B cell development and differentiation into plasma cells, followed by *CD79A*, whose surface protein contributes to the formation of the B cell receptor, and at third place *CD79B*, enhancing phosphorylation of CD79A. The top three specific genes in this cluster were the immunoglobulin-coding gene *IGHM*, Membrane Spanning 4-Domains A4A gene *MS4A4*, and the phosphatase and actin regulator 1 gene *PHACTR1*.

The three priority DEGs in cluster 3 included *GZMK* and *GZMA*, both of which produce serine proteases found in the cytoplasmic granules of cytotoxic lymphocytes and natural killer cells, along with *CCL4*, which serves as a chemoattractant for various immune cells, including natural killer cells and monocytes, at sites of inflammation or tissue damage. Among the top specific genes uniquely found in cluster 3, two genes were exclusively recorded, namely natural killer cell granule protein 7 gene *NKG7* and *TUBA4A* encoding for microtubule constituent.

Looking at cluster 4, the top three DEGs were T Cell Receptor Delta Constant gene *TRDC*, *XCL2*, which is predominantly expressed in activated T cells, and *XCL1* with chemotactic activity for lymphocytes. The corresponding top specific genes were *TNFRSF4*, whose protein is expressed on activated T cells, again *TRDC* and *TYROBP*, whose encoded protein may be associated with the killer cell immunoglobulin-like receptor family of membrane glycoproteins and act as an activating signal transduction element.

In cluster 5, the foremost DEGs encompassed *DERL3*, which likely participates in the degradation of misfolded glycoproteins within the endoplasmic reticulum, *IGHGP* encoding for Immunoglobulin Heavy Constant Gamma P, and *MZB1* involved in positive regulation of cell proliferation. The top three specific genes of cluster 5 were *PRDX4*, whose coding protein plays a regulatory role in the activation of the transcription factor NF-kappaB, *SDF2L1*, which is involved in chaperone cofactor-dependent protein refolding of misfolded proteins and is located in the endoplasmic reticulum, and *SEC11C* that catalyzes the cleavage of the N-terminal signal sequences of nascent proteins during translocation into the lumen of the endoplasmic reticulum.

In cluster 6, the first DEGs included *CPVL* encoding a carboxypeptidase, *LGALS2* encoding Galectin-2, and *HCK* playing an important role in the regulation of innate immune responses. The corresponding top specific genes of cluster 6 were *LYZ*, whose protein Lysozyme acts as an antimicrobial agent, *MS4A6A* encoding a member of the membrane-spanning 4A gene family, and *PLAUR* localizing and promoting plasmin formation.

Cluster 7 exhibited as top DEGs *PTCRA* forming the pre-T-cell receptor complex that regulates early T-cell development, *LILRA4* involved in limiting the innate immune responses to viral infections, and *IL3RA*, whose protein serves as a cell surface receptor for IL3 expressed on various immune cells, overseeing the production and differentiation of hematopoietic progenitor cells into lineage-restricted cells. Among the top specific genes within this cluster, *PTCRA* reappeared, followed by *SCT* encoding the hormone Secretin and *SERPINF1* encoding a neurotrophic protein representing a potent inhibitor of angiogenesis.

The corresponding heatmaps are depicted in Figure 4.

### 2.3. Conservation Analysis across Donors

In the following step, a conservation analysis was conducted across all donors, pooling all samples together to identify the top 10 conserved markers sorted by the most conservative significance assumption reflected by the combined *p*-value (max_pval). This *p*-value was computed using the highest *p*-value reported for a specific gene after running all individual analyses of each donor in each cluster. Genes were, however, called significant, if their combined minimum-based meta *p*-value was significant. Given that cluster 0 exhibited ubiquitous expression across all donors and predominantly harbored most of the PDL and gingival cells, while cluster 1 encompassed the majority of epithelial cells among all clusters, these two clusters were selected for identifying the genes within them that are conserved across all donors, thereby delineating the hallmark features of these clusters.

**Figure 5:**
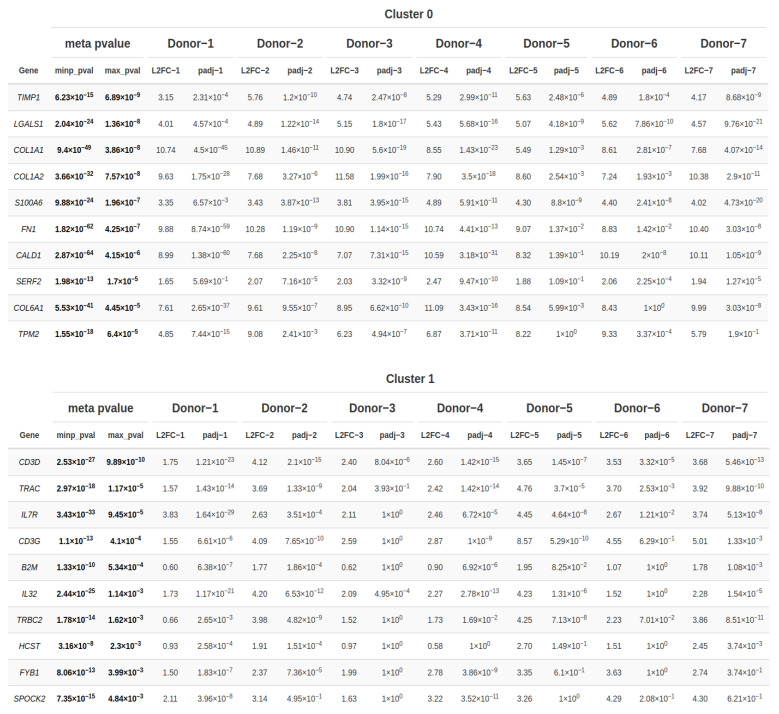
Conserved markers of clusters 0 and 1. The conservation analysis findings encompassing data from all seven donors reveal the identification of the top 10 conserved markers, ordered according to the most conservative significance assumption (max_pval) for both cluster 0 and cluster 1; Additionally, the liberal estimation of the combined *p*-value (minp_pval) is reported. Both meta *p*-values are highlighted in bold. The left-hand column enumerates the top 10 genes. Additionally, the results include the analysis of differential expression for each donor, where L2FC represents the log2 FoldChange of markers specific to a donor, and padj indicates the adjusted *p*-value.

As exhibited in Figure 5, in addition to the DEGs and top specific genes already identified, cluster 0 also harbored *FN1*, responsible for encoding fibronectin, a glycoprotein integral to the extracellular matrix playing pivotal roles in various physiological processes including tissue repair, embryogenesis, hemostasis as much as cellular migration and adhesion. Additionally, *CALD1*, encoding caldesmon, a protein pivotal in cytoskeletal regulation, was present in this cluster, alongside *SERF2* implicated in protein destabilization, and, finally, *TMP2*, whose protein β-tropomyosin is a striated muscle-specific coiled-coil dimer essential for stabilizing actin filaments and regulating muscle contraction.

Moving to cluster 1, alongside the previously identified DEGs and top specific genes, *IL7R*, encoding the Interleukin 7 Receptor, was notable. Interleukin 7, bound by this receptor, is inter alia produced by epithelial cells and represents a cytokine pivotal for the development of B and T cells. Furthermore, the cytokine interleukin 23 coding gene was among the top markers in cluster 1, which is inflammatory in nature and has been demonstrated to play a critical role in maintaining and expanding T helper type-17 cells. Additionally, cluster 1 encompassed *B2M*, integral to the class I major histocompatibility complex and decisive for presenting peptide antigens to the immune system, and *HCST*, encoding a transmembrane adapter protein pivotal for cytotoxicity induction against target cells expressing cell surface ligands by associating with KLRK1 to form an activation receptor. Lastly, *FYB1*, essential in signaling cascades within T-cells, and *SPOCK2*, encoding a member of a novel Ca(2+)-binding proteoglycan family, were also represented within this cluster.

### 2.4. Pseudotime Analysis

**Figure 6:**
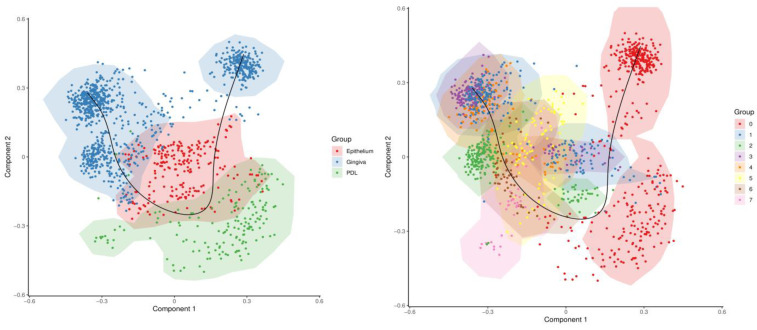
Pseudotime trajectory analysis. The pseudotime analysis results are depicted in a two-dimensional space delineated by principal components 1 (*x*-axis) and 2 (*y*-axis), derived through SCORPIUS’ dimensional reduction algorithm. On the left plot, the analysis encompasses the three tissue types under examination, namely PDL, gingiva, and epithelium, portraying a trajectory delineated by a black line, suggesting a possible developmental path or differentiation process. The analysis assumes a potential correlation between the progression of epithelium and PDL cells with gingival cells. Meanwhile, the right plot delineates cluster evolution, notably highlighting cluster 0 as distinctly separate from other clusters and situated at the terminal point of the pseudotemporal trajectory. This observation prompts the hypothesis that cluster 0 may denote a concluding developmental stage emerging subsequent to the earlier clusters. Color legends indicate the distinction between tissue types and clusters.

Finally, to elucidate the developmental progression of cells, a pseudotemporal trajectory inference was conducted using single-cell data. The results are plotted in a two-dimensional space defined by the principal components 1 and component 2 (Figure 6). This technique in single-cell transcriptomics arranges cells in a pseudo-temporal order based on their transcriptional profiles, shedding light on the dynamic developmental processes. In the left plot, the black line depicts the developmental trajectory, suggesting a potential correlation between the developmental progression of epithelium and PDL cells with gingival cells. It suggests that PDL cells may develop after epithelial and gingival cells, which could potentially also account for about one-third of the gingival cells sequentially emerging even after epithelial and PDL cells. In the right plot, the trajectory outlines the progression states of clusters in temporal sequence, indicating that the early formations could be represented by clusters 1–4, from which other clusters were then differentiated. Interestingly, these clusters were particularly prominent in donors with a low occurrence of cluster 0. According to the trajectory analysis, the PDL cell-dominated cluster 0 may represent a final developmental stage emerging from the prior clusters, supporting the trajectory analysis of tissue cellular development. However, this interpretation is hypothetical and requires additional analytical and biological validation.

## 3. Discussion

In this investigation, single-cell RNA sequencing was employed to delineate and characterize distinct cellular subpopulations within the predominant soft tissue components of the periodontium, namely epithelium, gingiva, and PDL. To the best of the authors‘ knowledge, this study marks the inaugural attempt to thoroughly investigate these three tissues, all from the same pool of healthy donors, concurrently and juxtapose them. The analysis unveiled a ubiquitous presence of epithelial and gingival cells across all clusters identified, while PDL cells were limited to two clusters, with the second cluster being exclusive to certain donors. Additionally, some cell clusters exhibited a consistent presence across all donors, indicating their intrinsic localization within the tissues. Contrarily, other clusters emerged selectively in certain donors, suggesting responsiveness to local environmental cues and contextual aspects. Notably, the consistent expression of particular genes within specific clusters across all donors revealed their critical functional involvement within these particular cell populations.

Given the clusters identified in this study, a noticeable division arises, allowing for their classification into four separate subgroups. The first subgroup exclusively includes cluster 0. Cluster 0 was consistently present in all donors analyzed and comprised the largest portion of cells, mainly consisting of PDL cells. Notably, the trajectory analysis of tissue cellular development showed a sequential pattern where PDL cells seemed to develop after epithelial and gingival cells. Cluster 0 signifies a unique population likely representing the final developmental stage emerging after the preceding clusters according to the pseudotime analysis. The prominent genes identified within this cluster are essential for upholding the integrity of PDL cells within the periodontium and ensuring their proper function and homeostasis. They play critical roles in regulating the cytoskeleton, maintaining cell structure, influencing matrix composition, orchestrating the cell cycle, facilitating adhesion, promoting proliferation, guiding migration, regulating differentiation, and facilitating wound healing processes.

While universally present across all donors, cluster 0 exhibited sparse representation in three donors that however contrasted with the pronounced prevalence of clusters 1, 2, 3, and 5, the first ones largely absent and the latter one undetectable in other donors. These four clusters combine to form the secondary subgrouping. Clusters 1, 2, and 3 stood out due to their abundance of immunological and inflammatory genes. In cluster 1, the predominant genetic components encoded proteins associated with inflammatory processes, major histocompatibility complex class II antigen presentation, and the maturation of T helper 17 cells. Additionally, essential elements of T cell receptor functionality, cellular proliferation, and signaling cascades were evident. Notably, this cluster showed a significant abundance of epithelial cells compared to the other clusters. Conversely, cluster 2 was marked by a predominance of gingival cells, with its prominent genetic signatures aligning with those indicative of B cell receptor modulation, developmental pathways, and intricate signaling mechanisms. In cluster 3, a genetic profile emerged that complemented the overall picture depicted by the preceding clusters, exhibiting markers for cytotoxic lymphocytes and natural killer cells, both pivotal players in immune surveillance and defense mechanisms. Moreover, this cluster showcased a gene associated with the secretion of chemoattractant cytokines, orchestrating the recruitment of immune cells to sites of inflammation and tissue damage, thus augmenting host responses to pathological insults. Cluster 5 revealed a distinct genetic pattern diverging from the immunological motifs seen in the other three clusters of this subgroup as it exhibited genes intricately engaged in vital biological processes of protein folding and unfolding aimed at correcting structural irregularities in misfolded proteins, thus safeguarding cellular integrity and function.

The third subgroup of clusters included clusters 4 and 6, which were consistently present in all donors and showed no significant differences in cell numbers, although with reduced amounts of cells. Cluster 4 displayed an immunological pattern marked by genes related to T cell function and adaptive immune responses, aligning with cluster 6, which showed genetic indicators associated with innate immune responses and antimicrobial activities.

The fourth subgroup was exclusively populated by cluster 7, which represents the second cluster com the last subgroup. The second cluster containing PDL cells alongside cluster 0 was notably found in donors 1 and 4. It was also detected in donors 5 and 7, but noticeably absent in all other donors. The genes associated with this cluster intricately govern processes of both T cells and innate immune cells.

The unique distribution and weighting of cells from various periodontal tissues within clusters, coupled with each donor’s distinct pattern of cluster representation, offer an expansive and new perspective on the genetic profiles and functional dynamics of these cells in diverse contexts. By employing the methodology to compare three distinct periodontal tissues from identical donors, this research unveils the complex interplay between cellular reactions and environmental influences within the periodontal microenvironment at the single-cell level, yielding profound insights into periodontal biology. Furthermore, by incorporating multiple donors, this study explores individual-specific differences in periodontal tissue composition, adding an additional dimension of complexity to the understanding of cellular dynamics.

All clusters except cluster 0 and cluster 5 suggest activated cellular cluster types involved in the immune response. Periodontal disease involves dense inflammatory infiltrates in connective tissue with T and B cell activation to control chronic inflammation and modulate osteoclastogenesis [16,17]. The activation of specific T and B cell subtypes is crucial in determining whether the inflammatory lesion stabilizes as chronic gingivitis or progresses to destructive periodontitis [18]. Previous studies have already shown that the gingival epithelium serves as a crucial mechanical barrier against bacterial invasion and contributes to the innate immune response in periodontal tissue, while its disruption of cell–cell interactions and ensuing inflammation are key factors in initiating periodontal disease [19,20]. Investigations have elucidated that epithelial cells mount an innate immune response upon encountering both nonperiodontopathic and periodontopathic bacteria by induction of antimicrobial peptides and interleukin-8, underscoring their function in host defense mechanisms [21].

Unexpectedly, the examination of the epithelial cells in this study showed a notable abundance of immune cell markers, suggesting the potential presence of a significant number of immune cells within the epithelial sample. Even though immune cells inhabit epithelial tissues, they possess unique functions and characteristics separate from epithelial cells. Typically, one would anticipate the presence of several epithelial cell markers as indicators of their identity. Nevertheless, the absence or minimal presence of these markers prompts further investigation, underscoring a crucial aspect concerning the cellular makeup of the samples. Emphasizing this point is essential as it could indicate a limitation in our cell typing methodology or point to a biological phenomenon within the epithelial tissue that warrants closer scrutiny. Given that the epithelial cell isolation method employed in this study is a well-established procedure in the literature, it is more likely to be the latter scenario. Consequently, our forthcoming research will focus on investigating this phenomenon in greater detail.

Prior investigations into the immunological attributes of periodontal soft tissue cells have elucidated that gingival cells undertake dual responsibilities, encompassing both the maintenance of gingival tissue architecture and the regulation of immune responses directed against oral pathogens [22,23]. While their activation assists in eliminating pathogens and resolving inflammation, uncontrolled activation can exacerbate inflammation and contribute to periodontitis, necessitating further investigation into therapeutic approaches targeting gingival cell interactions with pathogens and the immune system [24]. Furthermore, it is known that PDL cells also possess immunomodulatory properties and secrete proinflammatory cytokines, suggesting the possibility of further differentiation into immunocompetent cells capable of antigen presentation via MHC II [25,26].

In addition to the established knowledge concerning immunological functions within epithelial, gingival, and PDL cells, the findings of this study hold clinical relevance. This is particularly notable in elucidating the underlying mechanisms of periodontitis, despite the study’s exclusive utilization of tissue samples obtained from healthy human donors. The identification of characteristic genetic markers distinct for each tissue type and the enhancement of understanding of the immunological landscape in healthy tissues serves as a crucial foundation for discerning deviations that may predispose individuals to periodontitis, thereby facilitating earlier detection and intervention strategies in clinical settings.

It is important to emphasize that the present investigation was specifically directed toward a detailed analysis of samples procured from donors exhibiting complete periodontal health, rather than investigating periodontitis pathology. The primary objective of this preliminary study was to pioneer a comparison of the distinct genetic profiles intrinsic to the three primary periodontal soft tissue structures, namely gingiva, epithelium, and PDL. Additionally, a distinguishing characteristic of this research was the acquisition of samples from identical donors, enhancing the precision and validity of the data and inter-tissue comparisons.

Despite the constrained sample size and number of cells per sample, it is crucial to acknowledge the challenges inherent in procuring samples from individuals necessitating the excision of these three specific tissue types in parallel, with not all eligible patients consenting to participation. Notably, the inclusion of *n* = 7 surpasses the cohort sizes of preceding studies employing scRNASeq for periodontal tissue analysis, thereby encompassing a significantly larger sample set and enhancing statistical robustness in this regard [5,27,28,29]. Furthermore, while these prior investigations have focused solely on gingival tissue, this inquiry extends its purview to encompass the entirety of periodontal soft tissues. Emphasizing the primary objective of this research, it is pivotal to underscore that the analysis aimed to delineate the characteristics of three tissues in healthy donors, thus diverging from the mentioned studies predominantly contrasting periodontitis-affected individuals with healthy controls. By exclusively examining healthy donors, this study offers unique insights into the baseline attributes of these tissues, untethered from the confounding variables associated with periodontal disease pathology.

The results presented here provide valuable insights into the patterns of gene expression and cellular clusters within periodontal soft tissues. Nonetheless, it is important to note that these findings are based on a single method, which may have limitations. Therefore, it is crucial to interpret the conclusions considering the chosen methodology, while also being aware of potential biases and the likelihood of different results with alternative sequencing methods. Additionally, the relevance of the results may be influenced by the specific characteristics of the tissues studied, such as variations related to donors, which could affect gene expression and cellular reactions. Finally, this study did not include analyses of the individuals’ microbiome, a varied assembly of microorganisms essential for periodontal health, intricately influencing both health and disease [30]. Microbial imbalances, referred to as dysbiosis, are associated with periodontal disease, highlighting the crucial connection between microbial changes and periodontal health [31]. Therefore, it is important to carefully extend conclusions beyond the current dataset to ensure unbiased and thorough findings. Furthermore, validating identified genes and cell clusters across different methodologies is essential to confirm their functional significance. This study sets the groundwork for analyzing lineage relationships and characteristics of cellular subpopulations within PDL, gingiva, and epithelium, representing efforts that could ultimately lead to new therapeutic approaches in periodontal tissue regeneration. On the basis of these findings, future investigations may identify unique markers for epithelial, gingival, and PDL cells, offering prospects for innovative regenerative techniques and improved treatment strategies for periodontal health.

## 4. Materials and Methods

The study adhered to the ethical guidelines of the World Medical Association Declaration of Helsinki, obtaining informed consent from all human donors, and receiving Institutional Review Board approval from the Ethical Committee of the University of Bonn (reference number 029/08).

### 4.1. Isolation and Sample Preparation of PDL, Gingiva, and Epithelium

The primary tissues of human PDL, gingiva, and epithelium were sourced from periodontally healthy adult donors, comprising two males and five females with a mean age of 26.3 ± 11.2. These donors underwent extraction of erupted wisdom teeth (*n* = 7). Inclusion criteria for patients based on panoramic radiographs and clinical examinations comprised the absence of systemic issues or medications affecting oral health, no requirement for osteotomy to minimize the risk of periodontal ligament injury, fully developed root apices to prevent pulp tissue contamination during harvest, and immediate processing of samples post-extraction. The specimens were graciously provided by Private Practice Dr. Vollmar in Wissen, Germany, Private Practice Dr. Appel in Sankt Augustin, Germany, and the MEDECO Clinic in Bonn, Germany. The primary PDL was explanted from the middle third of the wisdom teeth root surfaces, while gingiva and epithelium tissues were obtained from excess tissues resected during the surgical extraction approach.

To obtain single-cell suspensions from PDL (*n* = 7), gingival (*n* = 7), and epithelial tissue (*n* = 7), enzymatic dissociations were conducted as outlined below.

For the PDL, extracted teeth were washed with DMEM medium, PDL cells were scraped off from the root surface, minced, and then incubated with 0.5 mL enzyme mix (12.5 μL collagenase/dispase and 5 μL DNase I) at 37 °C for 30 min.

For gingiva and epithelium, tissues were washed with DMEM medium (Thermo Fisher Scientific, Waltham, MA, USA), cut into 1–2 mm^3^ fragments, and incubated with 0.5 mL enzyme mix at 37 °C for 30 min. For gingiva, the enzyme mix consisted of 15 μL collagenase/dispase (Thermo Fisher Scientific) and 5 μL DNase I, (Thermo Fisher Scientific) while for epithelium, it was 5 μL collagenase/dispase and 5 μL DNase I.

Residual tissues were disaggregated by gentle flushing until single-cell suspensions were achieved. Each cell type was filtered through a 70 μm filter attached to 50 mL tubes. Filters were washed with 10 mL of medium and cells were centrifuged at 300× *g* for 5 min before resuspending them in 0.2 mL of medium. Viability testing and cell count analysis were performed using trypan blue and a hemacytometer.

Specimens were cryopreserved by adding 10% DMSO (Invitrogen, Carlsbad, CA, USA) into cell suspensions, storing samples at −80 °C for one day, and then transferring them to −150 °C.

### 4.2. Cell Hashing and Sorting from Single Cell Suspension

Thawed single-cell aliquots were mixed with 5 mL of medium and centrifuged at 500× *g* for 5 min. After removing the supernatants, cells were suspended in 100 μL of cell staining buffer (Biolegend, San Diego, CA, USA; cat # 420201). Then, 5 μL of human TruStain FcX™ (Biolegend, cat # 422301) Fc Blocking reagent was added to incubate for 10 min at 4 °C. An antibody pool was created using 1 µg of single-cell TotalSeq™-A025x anti-human Hashtag antibody (Biolegend). For optimal performance, the antibody pool was centrifuged at 14,000× *g* at 2–8 °C for 10 min before being added to the single cell suspension and incubated for 30 min at 4 °C. The cells were washed three times with 1 mL of cell staining buffer, spun for 5 min at 350× *g* at 4 °C, and finally resuspended in 500 μL of PBS (Thermo Fisher Scientific) in 1.5 mL tubes. PDL, gingiva, and epithelium tissue samples were combined from all patients and kept on ice. Before sorting, 1 μg/mL of propidium iodide (Thermo Fisher Scientific) was added and incubated at room temperature for 5 min. The cells were filtered through a 70 μm cell strainer or nylon meshes into a new tube and loaded into the cell sorter. Cell sorting was carried out using a BD FACSAria III high-speed cell sorter equipped with four lasers (405 nm, 488 nm, 561 nm, 640 nm) with 70 µm, 85 µm, and 100 µm nozzles. Living cells were collected in a new 1.5 mL tube containing 1 mL of PBS, pelleted by centrifugation, and then resuspended in fresh PBS.

### 4.3. Library Preparation

DNA samples were prepared for compatibility with the Illumina sequencer from 10× Genomics Inc., San Diego, CA, USA, using the Illumina 10× Genomics sequencing workflow to generate droplet-based scRNASeq data [32]. Initially, viability and separation efficiency of sorted cells were determined using the Countess II system (Thermo Fisher Scientific), followed by library preparation according to the manufacturer’s protocol (10× Genomics, Chromium Single Cell 3′ Reagent Kits v3 and CITE-seq) using 2500 cells. The CITE-seq protocol involved the addition of a specific HTO primer during cDNA amplification. In the subsequent cDNA purification step, the supernatant was used for HTO library preparation, while the bead-bound DNA was used for cDNA library preparation. Sequencing was conducted on the NovaSeq 6000 system with an SP flow cell, adhering to the 10× Genomics guidelines with read lengths of 28/8/0/91.

### 4.4. Sequencing and Demultiplexing

The libraries underwent pooling and sequencing on a NovaSeq sequencing system equipped with an SP flowcell, utilizing the following run specifications: 28 cycles for read 1, 91 cycles for read 2, and 8 cycles for index 1. The standard sequencing workflow of the instrument, encompassing template generation, imaging, and base calling with RTA, was employed. Subsequently, the binary sequencing data were demultiplexed and transformed into fastq format via bcl2fastq v2.20.0.422 (https://support.illumina.com/sequencing/sequencing_software/bcl2fastq-conversion-software.html; access date: 4 December 2021), ensuring no index-based mismatches (–barcode-mismatches 0) to accommodate the minimal edit distance between the two sample barcodes utilized in library preparation.

The sequencing yielded average sample sizes of 72 M, 253 M, and 80 M sequencing reads for PDL, gingiva, and epithelium, respectively, along with 56.5 M, 87.5 M, and 97.5 M reads for their corresponding HTO libraries. This deliberate variance in read distribution was predetermined based on a predefined pooling ratio, following a preliminary shallow sequencing experiment and subsequent saturation analysis. This analysis aimed to ascertain the optimal number of reads necessary to achieve maximal saturation at both the gene expression and HTO levels for each sample.

### 4.5. Cellranger Primary Analysis

The CellRanger analysis pipeline (https://www.10xgenomics.com/support/software/cell-ranger/downloads/previous-versions) (v3.1.0—access date: 23 April 2023) from 10× was used to perform the standard scRNA-Seq basic analysis. The pipeline workflow included the following steps: (1) alignment to the human genome reference sequence GRCh38 (preprocessed release 95—downloaded from the 10× Genomics website), (2) cell inference, (3) saturation analysis, and (4) various QC metrics. Starting from 1250, 2500, and 2500 initially set cells for PDL, epithelium, and gingiva, respectively, cell recovery rates of 21% (262 cells), 12.6% (314 cells), and 68.8% (1721 cells) were achieved. In addition, average sequencing depths of 276 K, 256 K, and 146 K reads per cell were received, respectively, which is significantly higher than the recommended average depth. Finally, for the HTO libraries, 5 K, 28.5 K, and 70.5 K usable HTO reads per cell were produced (10× recommendation is 5 K sequenced reads per cell). All samples reached a saturation level of ~98% in terms of sequencing depth.

### 4.6. Downstream Analysis with Seurat

#### 4.6.1. Data Integration and Cleaning

The Cellranger software was used to create single-cell gene expression matrices for downstream analysis, applying its standard filtering process to distinguish between GEMs (Gel bead-in-emulsion) containing cells and those either empty or containing ambient RNA. The Seurat package (v5.0.1) was then used to further process the filtered gene expression matrices (UMI counts per gene per cell) and de-multiplex the donor samples based on the hash information (HTO). For this purpose, an algorithm applying Seurat’s HTODemux function was used setting the quantile parameter to 99.9% and otherwise using standard parameters [33]. Samples were integrated using Seurat’s standard integration workflow and analyzed independently.

#### 4.6.2. Quality Checks

Analysis of the scRNAseq data started with initial quality control by assessing standard quality parameters to evaluate cell status in individual samples. Metrics comprising mitochondrial loads (mt.percent, which is the percentage of mt-genes in the detected transcriptome of a cell) indicating cell degradation and death, ribosomal load (rib.percent, which is the percentage of ribo genes in the detected transcriptome of the cell) serving as an indicator for cell cycle, the number of detected genes per cell (nFeature_RNA), and the number of transcripts (nCount_RNA computed by cellranger using unique molecular identifier (UMI) information) were employed to identify and eliminate low-quality cells distorting analyses. Cells exhibiting excessive mitochondrial or ribosomal loads constituting more than 25% or 50% of the detected transcriptome were excluded. Additionally, cells containing fewer than 150 genes were filtered out. Doublets, identified by an unusually high gene count in a single barcode, prompted the removal of cells with over 4000 genes. In summary, these cells were discarded to reduce the false positive calls of GEMs containing ambient RNA, especially debris and dying cells, typically reflected by high mitochondrial loads.

This refinement step, retaining only high-quality cells, was performed post-integration, as above mentioned poor-quality cells were called by cellranger and not regarded as empty GEMs or GEMs with ambient RNA. Therefore, they were initially included during integration to provide the input of the algorithm with a higher level of complexity. Subsequently, hashtagging (HTO) demultiplexing was conducted, with each sample barcoded and linked to HTO signals. The strongest signal was utilized as the donor identity using the HTODemux method, employing a quantile of 99.9%. Cell identities were categorized into singlets, cells with negative signals, and doublets. Singlets, representing single cells with statistically significant unique identity from one donor were further processed for downstream analysis. Negative cells were retained for downstream analyses to improve clustering performance and further procedures.

#### 4.6.3. Dimensionality Reduction and Clustering

First, a standard principal component analysis (PCA) was conducted, selecting the first 20 dimensions after visual inspection of the elbow plot showing principal components against standard deviation [34]. Next, the Uniform Manifold Approximation and Projection (UMAP) dimensionality reduction algorithm was employed to reduce the complexity of data by approximately maintaining both close and distant relationships found in high-dimensional space when mapping it to a lower-dimensional space [35]. This process allowed us to effectively visualize and understand the structure of various subpopulations within the dataset in two dimensions. The algorithm was executed using Seurat’s RunUMAP wrapper function, calling the implementation of the algorithm from the R-package uwot, and employing the specified dimensions and otherwise default parameters [15]. Subsequently, cell clustering was performed utilizing an SNN (Shared Nearest Neighbour) algorithm for modularity optimization via the FindClusters function [36]. This involved identifying the k-nearest neighbors of each cell, constructing the SNN graph, and optimizing a modularity function to delineate clusters at a given resolution. A clustering resolution of 0.25 was selected for integrated samples to prevent excessive clustering. Clustering was exclusively based on the biological characteristics of the cells.

#### 4.6.4. Differential Expression Analyses

Differential expression analyses between clusters were conducted using the “FindAllMarkers” function, employing the nonparametric Wilcoxon rank-sum test [37]. Seurat employs the test mentioned, utilizing the R-package presto by default, due to its clear conceptual framework and short run times, but especially its ability to handle the non-normality of counts commonly found in scRNA-Seq data. Conserved markers across donors were identified using the FindConvervedMarkers function, which calculates the differential markers for each specific cluster and donor within the cluster compared to all other clusters. It then computed a combined *p*-value, termed meta-*p*-value, using the standard minimum method from the R package metap. This method selected the minimum *p*-value across all analyses to estimate a corrected combined *p*-value reflecting the overall significance considering the collective probability of obtaining at least one significant result from the multiple tests. Along with the minimum *p*-value, a conservative meta *p*-value (max_p_val)) that was in contrast to the former *p*-value calculated based on the highest *p*-value reported by the individual analyses of each donor. Both *p*-values were reported by default by Seurat and used to assess the significance of calls. In the method applied here, the markers were sorted by max_p_val, thus selecting top markers based on this *p*-value in order to discuss the most confident markers based on the dataset, while the minimum_p_val significance metric was used to call a gene significant. In this way, both very conservative and less conservative estimations were considered. Reliable conclusions were drawn for each cluster only from donors contributing more than two cells within the cluster. To identify genes with high specificity, the discrimination analysis method implemented with GeneSorteR was applied [38].

### 4.7. Pseudotemporal Trajectory Inference

The SCORPIUS (v1.0.9) package was used for pseudotemporal trajectory inference of single-cell data to determine the sequential arrangement of cells along developmental trajectories [39]. This method was reported to be among the top-performing trajectory inference methods and thus superior to most alternative approaches [40]. In brief, using the SCORPIUS method, PCA was utilized for dimensionality reduction, followed by the inference of a minimal spanning tree (MST) for trajectory construction. SCORPIUS employed the Spearman correction for computing distances between points in a low-dimensional space. To construct the trajectory, dimensionality reduction was performed with the “reduce_dimensionality” method of the package, selecting the Spearman correlation for the “distance” parameter and selecting three dimensions. Then, the “infere_trajectory” method was executed with the default parameters. Finally, the trajectory was visualized using the “draw_trajectory_plot” method.

## 5. Conclusions

In this investigation, advanced single-cell RNA sequencing (scRNA-Seq) technology was employed to analyze the genetic landscape of periodontal tissues, specifically focusing on epithelium, gingiva, and PDL. Our analysis revealed intricate cellular compositions of these tissues, highlighting distinct genetic profiles and cellular clusters. Notably, inherent subpopulations as well as those influenced by environmental cues were identified, indicating the dynamic nature of periodontal tissues.

This discovery enhances our understanding of the cellular organization of periodontal tissues and emphasizes the significant role of environmental factors in shaping their characteristics. By uncovering these phenomena, our research establishes a foundation for future investigations aimed at elucidating the underlying mechanisms. These insights are poised to advance our knowledge of the fundamental dynamics of periodontal tissues and pave the way for further exploration in this field. 

## Data Availability

The raw data supporting the conclusions of this article will be made available by the authors on request.
